# Overlapping genes of *Aedes aegypti*: evolutionary implications from comparison with orthologs of *Anopheles gambiae* and other insects

**DOI:** 10.1186/1471-2148-13-124

**Published:** 2013-06-18

**Authors:** Susanta K Behura, David W Severson

**Affiliations:** 1 Eck Institute for Global Health, Department of Biological Sciences, University of Notre Dame, Notre Dame, IN 46556, USA

**Keywords:** Gene rearrangement, Culicidae, Genome evolution, Positionally overlapping genes, Negative selection

## Abstract

**Background:**

Although gene overlapping is a common feature of prokaryote and mitochondria genomes, such genes have also been identified in many eukaryotes. The overlapping genes in eukaryotes are extensively rearranged even between closely related species. In this study, we investigated retention and rearrangement of positionally overlapping genes between the mosquitoes *Aedes aegypti* (dengue virus vector) and *Anopheles gambiae* (malaria vector). The overlapping gene pairs of *A*. *aegypti* were further compared with orthologs of other selected insects to conduct several hypothesis driven investigations relating to the evolution and rearrangement of overlapping genes.

**Results:**

The results show that as much as ~10% of the predicted genes of *A*. *aegypti* and *A*. *gambiae* are localized in positional overlapping manner. Furthermore, the study shows that differential abundance of introns and simple sequence repeats have significant association with positional rearrangement of overlapping genes between the two species. Gene expression analysis further suggests that antisense transcripts generated from the oppositely oriented overlapping genes are differentially regulated and may have important regulatory functions in these mosquitoes. Our data further shows that synonymous and non-synonymous mutations have differential but non-significant effect on overlapping localization of orthologous genes in other insect genomes.

**Conclusion:**

Gene overlapping in insects may be a species-specific evolutionary process as evident from non-dependency of gene overlapping with species phylogeny. Based on the results, our study suggests that overlapping genes may have played an important role in genome evolution of insects.

## Background

Gene rearrangement is one of the necessary ingredients of genome evolution. Several well studied mechanisms such as chromosomal inversions, translocations, duplications and transpositions are known to have important roles in genomic rearrangement events [1-4]. Reshuffling of genomic DNA by gross chromosomal rearrangements generally involves a number of genes that undergo positional relocation in the genome. In addition to such large scale genomic rearrangements, genomic rearrangements at small scale levels facilitate relocation of genes which are otherwise positionally overlapping in a genome [5]. It has been suggested that transposition mechanisms may contribute to such gene arrangements [1,2,6,7], but the functional and evolutionary significance of such events is largely unknown.

Positional overlapping between genes is a common structural feature of prokaryote and mitochondria genomes [8-10]. However, overlapping genes have also been identified from whole genome sequences of several eukaryotes such as fruit fly, zebrafish, human, chimpanzee, orangutan, marmoset, rhesus, cow, dog, mouse, rat and chicken [11-13]. Studies show that overlapping genes in eukaryotes are extensively rearranged even between closely related species [5,12,14-16]. Bhutkar *et al*. 2007 [5] compared overlapping genes of *Drosophila melanogaster* and *Anopheles gambiae* with *Apis mellifera* (honey bee) and suggested that relocalization of overlapping genes may have played a significant role in genome evolution of these insects. Although several other insect genome sequences are now available, overlapping genes of most of these insects have not been studied.

The present study is an effort to investigate overlapping genes of *Aedes aegypti*, the primary global vector of dengue virus, in a comparative manner with those of *A*. *gambiae*, a major vector of malaria in subSaharan Africa. Understanding genome structure of these mosquitoes has become one of the major interests among insect vector biologists. At present, the draft genome sequences for three mosquito species have been completed [17-19]. These projects (http://www.vectorbase.org) have provided new insights on structure, function and evolution of mosquito genes, thus furthering our ability to study mosquito-parasite or mosquito-virus interactions at the molecular level [20-23].

We identified positional overlapping of genes at the whole genome level in *A*. *aegypti* and studied structural differences and evolutionary features by comparisons with orthologous genes of *A*. *gambiae* and other selected arthropod genomes. The primary aim was to test several common hypotheses relating to rearrangement of overlapping genes and determine factors that may have a role in relocalization of overlapping genes in insects. The results of our investigation show that positional overlapping among genes is a species specific evolutionary process as evident from non-dependency of gene overlapping with species phylogeny, and also show that specific factors, such as introns and repeat sequences, are significantly associated with retention/rearrangement of overlapping genes in mosquitoes. Based on these results, our study suggests that overlapping genes may have played an important role in genome evolution among insects.

## Methods

### Official gene sets and extraction of overlapping gene pairs

The overlapping gene pairs of *A*. *aegypti* and *A*. *gambiae* were identified in a genome-wide manner based on the coordinates of gene boundaries of official gene sets annotated from the genome assemblies. The other mosquito genome sequence for *Culex quinquefasciatus* was not used for this purpose because of differences in gene annotation of this species compared to *A*. *aegypti* or *A*. *gambiae*. That is, while nearly equally percentages (~60%) of the official gene sets of *A*. *aegypti* as well as *A*. *gambiae* have been annotated for gene boundaries that incorporated the 5’ and 3’ untranslated regions, only 15% of the *C*. *quinquefasciatus* genes have been annotated in this manner. Thus, incorporating *C*. *quinquefasciatus* could have produced biased results in the genome-wide comparison of overlapping gene pairs between *A*. *aegypti* and *A*. *gambiae*. However, we have used orthologs of *A*. *aegypti* overlapping gene pairs in *C*. *quinquefasciatus* and other selected insect species such as *Drosophila melanogaster*, *Apis mellifera*, *Pediculus humanus*, *Bombyx mori* and *Acyrthosiphon pisum* to determine if they are also localized in overlapping positions in the respective genomes. For genome-wide comparison of overlapping genes, the predicted gene sets of *A*. *aegypti* (AaegL1.1) and *A*. *gambiae* (AgamP3.4) along with coordinates of genes in the reference genome were downloaded from VectorBase (http://www.vectorbase.org/GetData/). The one-to-one orthologous genes (OrthoDB5; http://cegg.unige.ch/orthodb5) were compared to determine if they were also present in overlapping gene pairs across multiple genomes. To determine the relative position of the orthologous genes, the official gene lists along with their start and end positions in the genome sequences of the other six insects (*C*. *quinquefasciatus*: CpipJ1, *D*. *melanogaster*: BDGP 5, *A*. *mellifera*: Amel_2.0, *P*. *humanus*: PhumU1, *B*. *mori*: SilkDB V2.0 and *A*. *pisum*: Acyr2) were downloaded from either VectorBase (http://www.vectorbase.org/) or the SilkDB database (http://www.silkdb.org) or the ‘Ensembl Metazoa 10’ data sets at http://www.biomart.org.

### Intron analysis

To determine if introns have an association with overlapping between genes, orthologous genes were categorized as intronless and intron-containing genes for overlapping and non-overlapping pairs in the *A*. *aegypti* and *A*. *gambiae* genomes. The exon structures predicted for *A*. *aegypti* and *A*. *gambiae* genes (obtained from Biomart.org) were used to classify genes into single exon genes (intronless) and multi exon genes (intron-containing). The number of introns in each gene was determined from the number of exons annotated in the genes. The 2x2 contingency analysis of counts of the intronless and intron-containing genes of both categories (overlapping/ non-overlapping) was performed using Yates Chi square tests to determine significance of association between introns and gene overlapping.

### Transcriptional analysis of overlapping genes

The expressed sequence tags (EST) of *A*. *aegypti* and *A*. *gambiae* mosquitoes used in this study were largely generated in conjunction with the individual genome sequencing projects (http://www.vectorbase.org). These ESTs were used to assist in the annotation of the official gene sets of the two mosquitoes. We used these ESTs to investigate expression patterns associated with the overlapping gene pairs. To further confirm correspondence of ESTs with overlapping gene pairs, we performed reciprocal BLAST analyses described as follows. The EST sequences were used to generate a local BLAST database and then searched by BLASTN with the sequences of overlapping genes. The EST ‘hits’ that had an e-value = 0 were used again as queries in another BLASTN search against all predicted gene sequences. If the reciprocal hits matched the same gene that was used as a query in the first BLAST, it was considered that the EST corresponded to that gene. Apart from analyzing the EST data, we also analyzed previously performed microarray expression data of *A*. *aegypti*[23] to determine expression patterns of the overlapping gene pairs. The *A*. *gambiae* microarray expression data was obtained from Baker *et al*. 2011 study [24]. The expression data of these studies [23,24] are publicly available with Gene Expression Omnibus (GEO) accession # GSE16563 and GSE21689 at http://www.ncbi.nlm.nih.gov/geo/. The Spearman’s rank correlation test was conducted to ascertain whether the overlapping gene pairs had significantly correlated expression levels throughout the genome.

### Identification of microsatellites in overlapping genes

In order to determine if there is a significant association of microsatellites with retention or rearrangement of overlapping gene structures between *A*. *aegypti* and *A*. *gambiae*, we identified microsatellite sequences within the gene pairs in both genomes. SciRoKo, a simple sequence repeat (SSR) identification program [25], was used to detect both perfect and imperfect mono-, di-, tri-, tetra- and hexa-nucleotide repeats using the default parameters (mismatch, fixed penalty = 5). The repeats with more than 3 consecutive mismatch sites were excluded. The genes where one or more sites were ambiguous nucleotides (such as ‘N’s) were not used to report microsatellites. The length of orthologous genes may vary (primarily because of introns) that may contribute to varying amounts of microsatellite sequences in the orthologous gene copies. So, instead of comparing the absolute amounts of microsatellite sequences, their relative amounts were compared. The relative amounts were obtained from the total amount of microsatellites of genes normalized with the alignment length (common DNA sequences) of the orthologous genes between *A*. *aegypti* and *A*. *gambiae*.

### Statistical and computational analyses

All statistical analyses were performed using the *R* statistical program. The *p*-value < 0.05 was considered statistical significance in all tests unless stated otherwise. Cluster analyses of gene pairs based on overlapping or non-overlapping structures across genomes were based on average correlation of city-block distance estimated using the Cluster3 program [26]. The phylogenetic analyses were performed by neighbor-joining method using MEGA4 [27]. The evolutionary distances were in the units of the number of base substitutions per site; and they were calculated using the Maximum Composite Likelihood method [28]. The Mantel procedure [29] was used to perform linear regression between matrices where the dependent matrix (representing 0 for non-overlapping and 1 for overlapping) was permutated 1000 times to test significance of the observed correlation with the independent matrix (that represented presence or absence of orthologs of overlapping gene pairs of *A*. *aegypti*) in the genomes used for comparison. The multi Mantel procedure was performed using an algorithm developed by Dr. Liam J. Revell (URL: http://anolis.oeb.harvard.edu/~liam/programs/). Maximum likelihood methods described elsewhere [30,31] were used to estimate the log likelihoods of models assuming either dependency or non-dependency of gene phylogeny with the discrete variation of gene traits (i.e. overlapping or non-overlapping localization in the respective genomes). The likelihood ratio tests were conducted to infer statistical significance of these two models. A binary logit model was developed to test marginal effects of the rates of synonymous (dS) and non-synonymous (dN) mutations in the orthologous gene pairs between *A*. *aegypti* and other select insect genomes (*A*. *gambiae*, *C*. *quinquefasciatus*, *D*. *melanogaster* and *P*. *humanus*). While each of the gene pairs (n =19) were localized in an overlapping manner in the *A*. *aegypti* genome, the orthologous genes showed variation in relative localization (overlapping = 1or non-overlapping = 0) in other species. The dN and dS values of orthologous genes were obtained from metazoan genes database at http://www.Biomart.org. A generalized linear model (described in detail in results section), fitting the dependent variable (0 or 1) and independent variables (dN and dS values for both genes), was used in *R* to estimate the logit coefficients.

## Results and discussion

### Identification of overlapping genes

A total of 761 and 565 overlapping gene pairs were identified in the assembled genomes of *A*. *aegypti* and *A*. *gambiae*, respectively (Additional file 1). They represent 8-10% of the annotated genes of the two mosquitoes. The frequencies of overlapping genes of *A*. *aegypti* and *A*. *gambiae* mosquitoes are within the range of overlapping gene frequencies reported in other eukaryotes [32,33]. More than two genes (overlapping gene clusters) were also found in overlapping locations in both genomes, with the majority of these overlapping gene clusters containing no more than three genes (21 clusters in *A*. *aegypti* and 19 in *A*. *gambiae*). These overlapping clustered genes constituted only a minor portion (less than 3%) of the total number of overlapping genes in either of the two genomes. Because of low frequency and also for simplicity of analysis, we have not included the gene clusters in our investigation. All the analyses performed in this study were based on overlapping gene pairs.

### Orthology of overlapping genes between *A*. *aegypti* and *A*. *gambiae*

**Figure 1:**
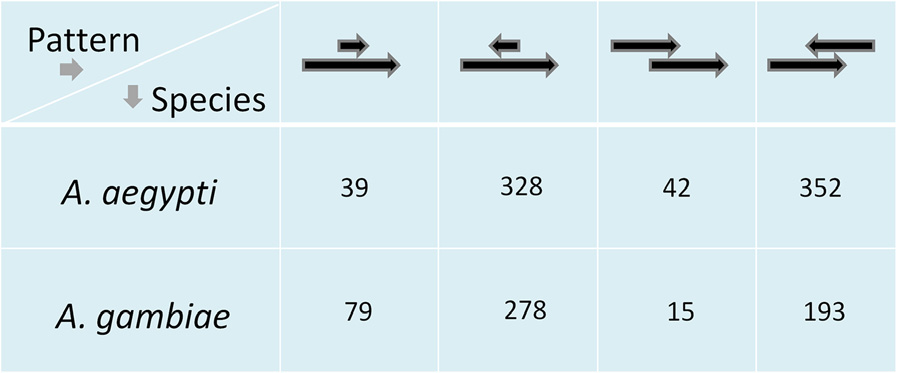
**Patterns localization of E/H and P/O gene pairs in the genomes of three mosquito species.** The gene pair counts and relative orientations are also shown.

**Figure 2:**
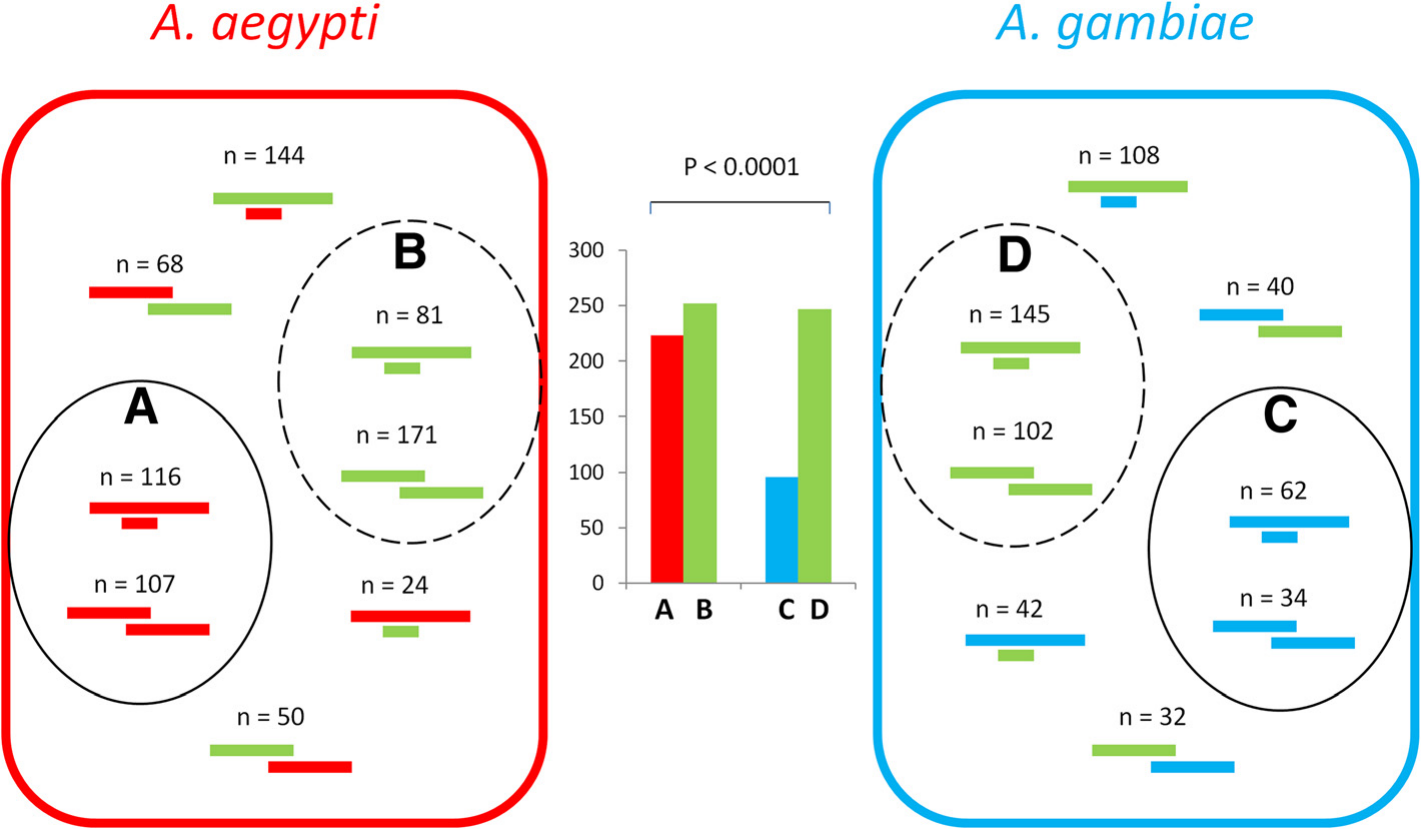
**Orthology (one-to-one) of overlapping gene pairs between *A. aegypti *and *A. gambiae.*** Genes have been color coded to represent orthologous relationships between the two species: red color represents *A*. *aegypti* and blue color represents *A*. *gambiae*. The one-to-one orthologous gene pairs that are localized in positionally overlapping manner in both the genomes are indicated by green color. The number of gene pairs is shown for each group. The circles **A** and **C** represent the young-young gene pairs, circles **B** and **D** represents the old-old gene pairs, and the gene pairs outside the circles represent young-old gene pairs in each species. The 2x2 contingency tests based on the count statistics of young-young and old-old genes between *A*. *aegypti* and *A*. *gambiae* (i.e. the **A**, **B**, **C** and **D** gene groups) shows that there is a significant bias in the distribution of these genes between the two species (shown as the column graph in the middle). In this graph, the y-axis shows the number of gene pairs corresponding to the four gene groups; and the gene groups are represented by the x-axis. The columns are color coded same manner as that of the gene groups (**A**, **B**, **C** and **D**).

**Figure 3:**
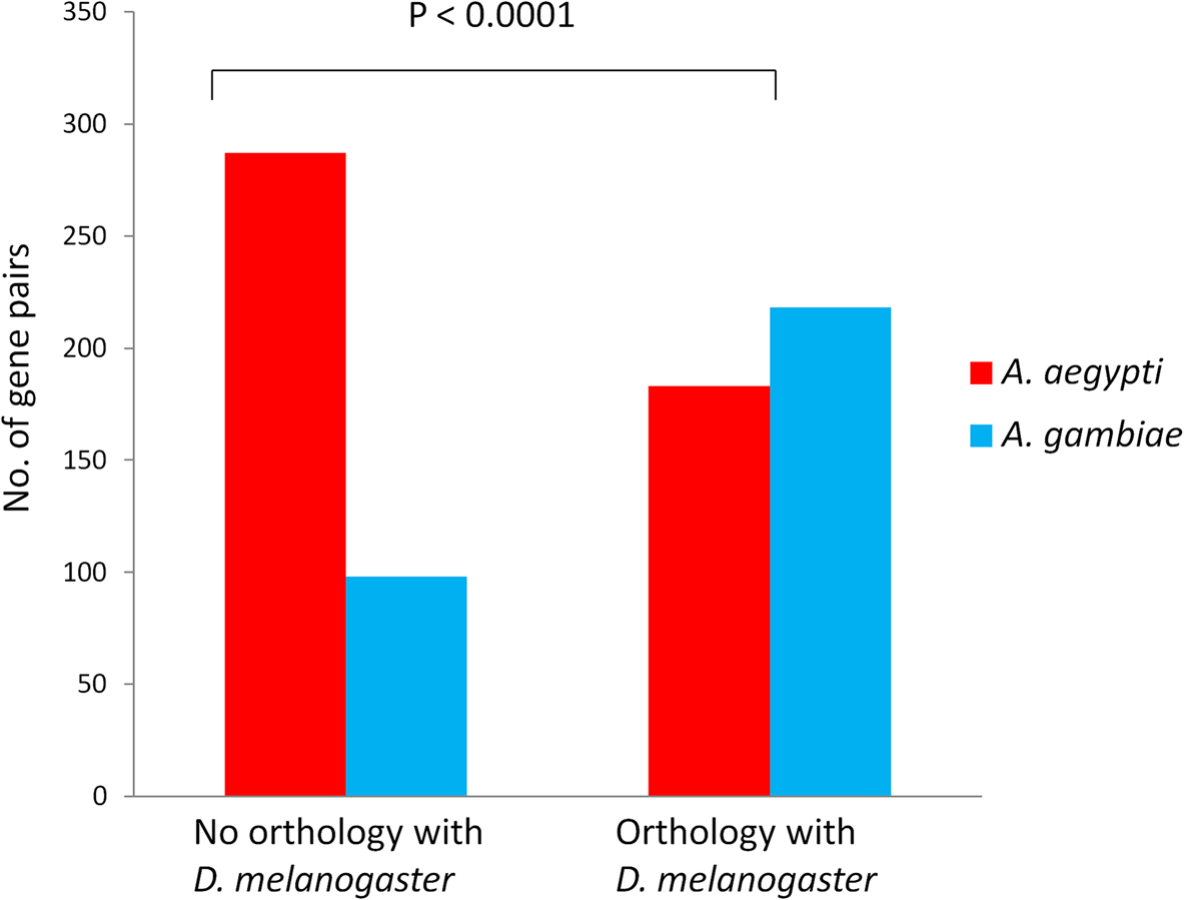
**Significant variation in the number of overlapping gene pairs of *A. aegypti *and *A. gambiae *based on presence or absence of orthology in *D. melanogaster *genome.** Y-axis shows number of gene pairs and x-axis shows whether the pairs have or don’t have orthology of *A*. *aegypti* and *A*. *gambiae* genes in the *D. melanogaster* genome. The *p*-value shows significance by Chi square test. It shows that the ancestral genes (where orthology is evident in *D*. *melanogaster*) are comparable in numbers between *A*. *aegypti* and *A*. *gambiae* whereas the young gene pairs vary significantly between the two species suggesting that the young genes are major contributors to the positional overlapping among genes.

In *A*. *aegypti* as well as in *A*. *gambiae*, the overlapping gene pairs are localized either in nested form (one gene embedded within another gene, the embedded/host genes or E/H genes) or in partially overlapping form (henceforth abbreviated as P/O genes) as shown in Figure 1. Irrespective of whether gene pairs are in E/H or in P/O form, they are predominantly localized in opposite orientation to each other. Three possible patterns of evolution emerged from comparing the orthologs of overlapping genes between the two species: 1) the gene pairs are orthologous between the two species (‘old-old gene pairs’), 2) the gene pair is specific to the species (lack of orthology in the other species, ‘young-young gene pairs’), and 3) one of the genes is specific to the species and the other is common between the two species (‘young-old gene pairs’) (Figure 2). The number of young-young gene pairs (shown as circle A and C in Figure 2) and the old-old gene pairs (shown as circle B and D in Figure 2) vary between the two species. The 2x2 contingency tests based on the count statistics of old-old and young-young gene pairs between *A*. *aegypti* and *A*. *gambiae* (the A, C, B and D gene groups) shows that there is a significant bias in the distribution of these genes between the two species. It clearly shows that nearly the same number (~ 250) of orthologous gene pairs (old-old pairs) are present in overlapping manner in both the species, whereas a significantly larger number of *A*. *aegypti* specific genes (young-young pairs, circle A) are in overlapping position compared to *A*. *gambiae* (circle C). This shows that the young-young gene pairs show significant variation in overlapping patterns between these mosquitoes. When the *A*. *aegypti* and *A*. *gambiae* overlapping genes were compared with *D*. *melanogaster* orthologs, consistent patterns were observed (Figure 3). The data in Figure 3 shows that the old-old gene pairs (genes that have orthologous copies in *D*. *melanogaster* genome) are comparable in numbers between *A*. *aegypti* and *A*. *gambiae* whereas the young-young gene pairs (genes that lack orthologous copies in *D*. *melanogaster* genome) vary significantly between the two species. Consistent with the results shown in Figure 2, these results also suggest that young genes are major contributors to the positional overlapping of genes in these species.

### Rearrangement of overlapping genes

One of the common hypotheses about positional overlapping of genes is that selection acts against the retention of gene overlap between genomes [14,33]. If the above hypothesis is correct, we expect that overlapping genes should be extensively rearranged between *A*. *aegypti* and *A*. *gambiae*. To test that expectation, the orthologous (one-to-one) copies of overlapping gene pairs were compared between the two species (Additional file 2). It was found that only 139 of the total 499 orthologous gene pairs are localized in overlapping manner in both the genomes. The other 360 gene pairs are localized in overlapping manner in one genome but in non-overlapping manner in the other (Table 1) suggesting that only a fraction of overlapping genes are retained across genomes. To determine if retention or rearrangement of overlapping localization of genes between *A*. *aegypti* and *A*. *gambiae* may be associated with loss or gain of terminal exons of genes, we investigated several gene pairs that contain multiple exons in the orthologous gene pairs (Additional file 3) and found no discrepancy in annotation of first and last exon of any gene pair between the two species.

**Table 1:** **Number of one-to-one orthologous gene pairs which are localized either in overlapping or non-overlapping manner relative to each other between the *A***. ***aegypti and A***. ***gambiae *genomes**

**Localization pattern**	**Number of gene pairs**
E/H in both *A*. *aegypti* and *A*. *gambiae*	75
P/O in both *A*. *aegypti* and *A*. *gambiae*	54
E/H in *A*. *aegypti* but P/O in *A*. *gambiae*	3
P/O in *A*. *aegypti* but E/H in *A*. *gambiae*	7
E/H in *A*. *aegypti* but non-overlapping in *A*. *gambiae*	43
P/O in *A*. *aegypti* but non-overlapping in *A*. *gambiae*	140
Non-overlapping in *A*. *aegypti* but E/H in *A*. *gambiae*	103
Non-overlapping in *A*. *aegypti* but P/O in *A*. *gambiae*	74

The overlapping gene pairs where one gene is embedded in another are represented as E/H pairs. The gene pairs which are localized in partially overlapping manner with each other are represented as P/O gene pairs.

**Figure 4:**
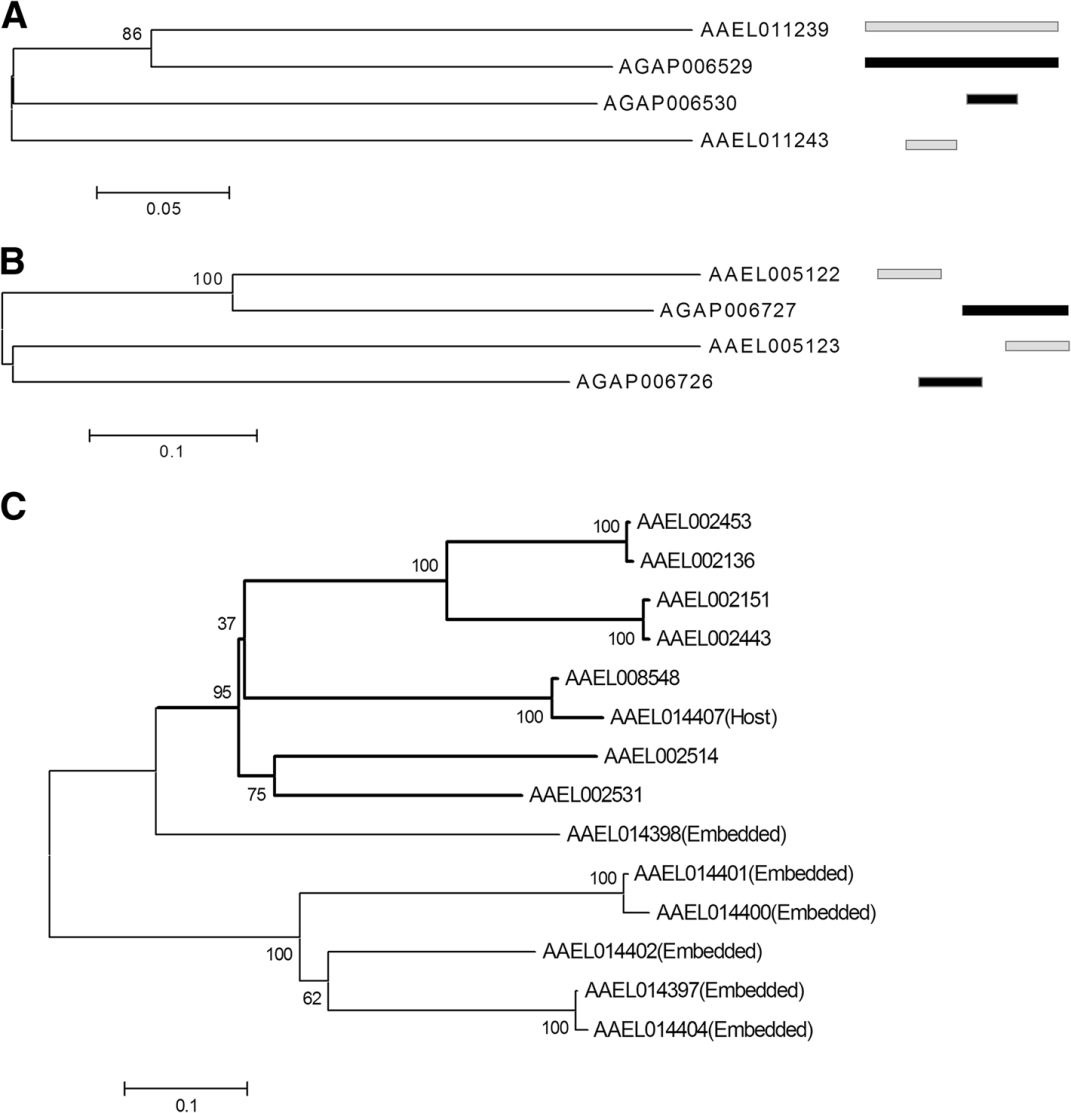
**Phylogenetic (neighbor-joining trees) relationship among pairs of genes of *A. aegypti *(gene ID begins with AAEL; gene structural pattern shown as light color bars next to branches) and *A. gambiae *(gene ID begins with AGAP; gene structural pattern shown as dark color bars next to branches).** The gene pairs in each case are paralogous to each other within species and at the same time they are orthologous to each other between the two species. They either retain positional overlapping structure in both the genomes (**A**) or show overlapping in one but non-overlapping in the other (**B**). The tree shown in (**C**) represents phylogenetic relationship of the host gene (AAEL014407) with paralogous copies in the *A*. *aegypti* genome which are either embedded (within AAEL014407) or non-embedded (located outside AAEL014407). The genes that are embedded are marked so within brackets. The scale for branch length is shown below each tree.

Additionally, we performed specific case studies of retention or rearrangement between the two species. A short-chain dehydrogenase gene (AAEL011239) acts as the host of another protein coding gene AAEL011243 (a paralog of AAEL011239) in *A*. *aegypti*. It was found that the corresponding orthologs in *A*. *gambiae* genome are also localized in E/H form (Figure 4A). In contrast to this, the gene (AAEL005122) of *A*. *aegypti*, that codes for a carboxylesterase, is localized in the genome in non-overlapping manner with one of its paralogs (AAEL005123) whereas the ortholog in *A*. *gambiae* (AGAP006727) is localized in P/O manner with the paralog AGAP006726 (Figure 4B). These genes associated with retention or rearrangement of positional localization in both the genomes are known to have significant changes in expression during blood feeding, growth and development of these mosquitoes [34-36]. In another case, we identified multiple genes that are embedded within intron sequences of a single host gene in *A*. *aegypti*. The gene AAEL014407 that putatively codes for the protein B-cell lymphoma/leukaemia (11A extra long form) harbors 6 paralogous genes within one intron along with several other paralogous genes that are localized in non-overlapping manner to AAEL014407 (Figure 4C). Importantly, these genes have been reported to be expressed in *A*. *aegypti*[36]. Moreover, the phylogenetic tree (Figure 4C) shows that the embedded genes tend to cluster together and are phylogenetically distinct from the genes located outside the host genes. This suggests a contrasting rate of evolution of embedded versus non-embedded paralogous copies of the gene AAEL014407 *in A*. *aegypti*.

### Gene overlapping is phylogeny independent

**Figure 5:**
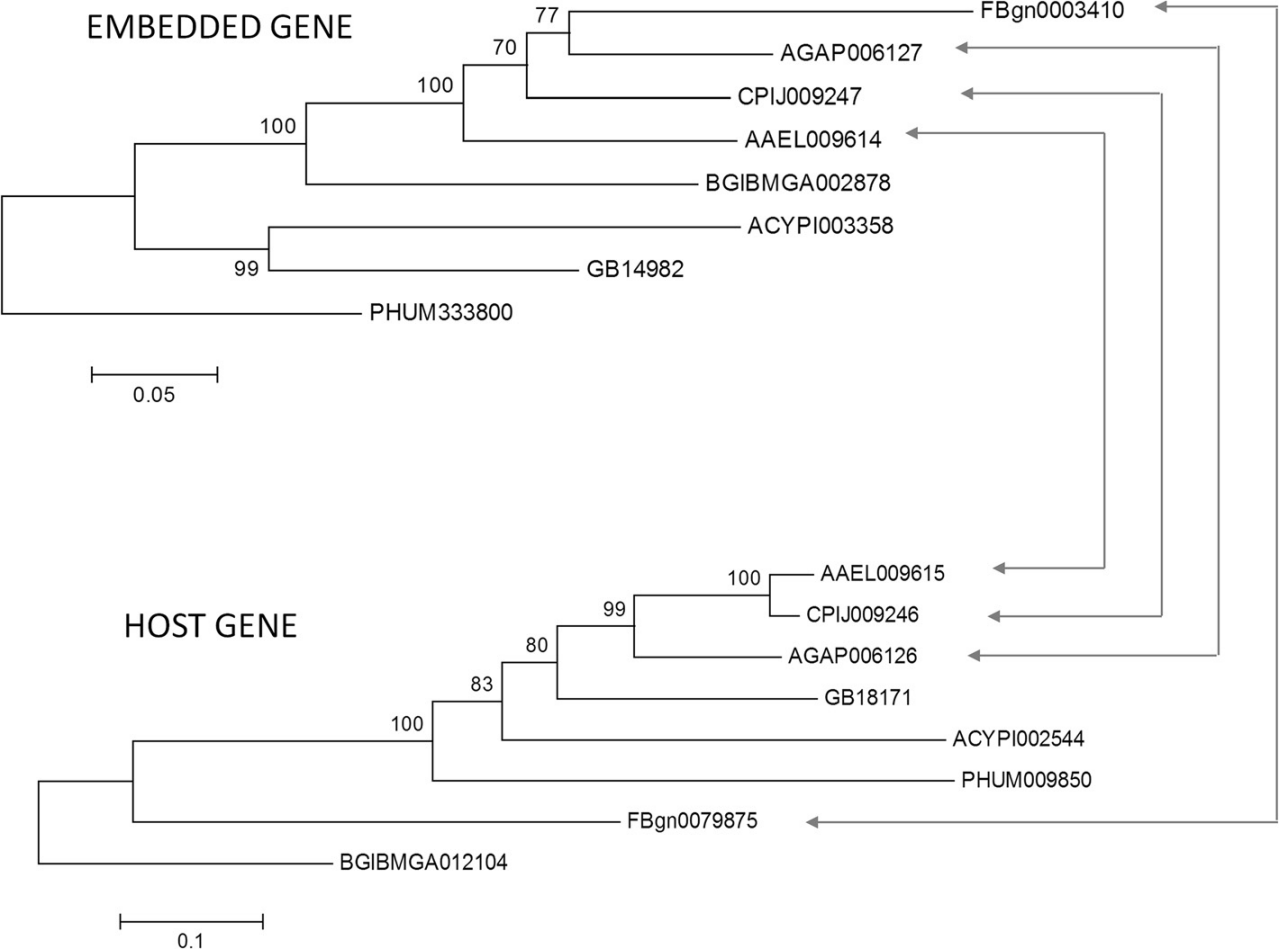
**Neighbor-joining phylogenetic trees of a representative gene pair among different insect species.** The positional overlapping (one gene embedded in another), if present in a species, is indicated by arrowed lines connecting the corresponding genes between the two phylogenies. The alphabetic letters associated with gene IDs shown in the tree correspond to the species as follows: AAEL- *A*. *aegypti*, AGAP- *A*. *gambiae*, CPIJ- *C*. *quinquefasciatus*, FBgn- *D*. *melanogaster*, GB- *A*. *mellifera*, PHUM- *P*. *humanus*, BGIBMGA- *B*. *mori* and ACYP- *A*. *pisum*. The scale for branch length is shown below each tree.

Having observed that orthologs of overlapping genes are extensively rearranged between species, we hypothesized that phylogenetic relationship has no correlation with the localization pattern (overlapping or non-overlapping) of orthologous genes across species. If this is true, one would expect that gene overlapping occurs as a trait which should be independent of species phylogenies. To test this hypothesis, we performed ‘discrete analysis’ of gene overlapping across phylogeny using maximum likelihood method [30] to determine if presence or absence of gene overlapping is correlated with phylogeny among species. Seven insect species (selected based on varying phylogenetic relationships with *A*. *aegypti*) were compared for orthology analysis with *A*. *aegypti* overlapping genes: *A*. *gambiae* (malaria mosquito), *C*. *quinquefasciatus* (southern house mosquito), *D*. *melanogaster* (fruit fly), *A*. *mellifera* (honey bee), *P*. *humanus* (body louse), *B*. *mori* (silkworm) and *A*. *pisum* (pea aphid). The results of this analysis showed that the likelihood estimates of the null model (independence of gene overlapping with phylogeny) consistently lack statistical significance when tested against the alternate model (dependency of gene overlapping with phylogeny) (Table 2). It clearly shows that there is no apparent relationship of positional overlapping with the phylogeny of the species. To illustrate this, a representation of phylogeny and gene overlapping pattern is shown in Figure 5 for orthologs of *A*. *aegypti* gene pair AAEL009614-AAEL009614 (E/H gene pair) among seven other insect species. It shows that retention or rearrangement of orthologous genes lacks correspondence with the phylogenetic relationships between species.

**Table 2:** **Positional overlapping/non-overlapping patterns of orthologs of *A***. ***aegypti *gene pairs in 7 other insect species were compared with species phylogeny**

**Orthologs of gene pairs**	**Model dependent**	**Model independent**	**Log likelihood difference**	***p***-**value**
AAEL00241/ AAEL000233	−6.28154	−6.74766	0.466125	0.976714
AAEL006054/ AAEL006056	−3.32599	−3.49681	0.170826	0.996554
AAEL008942/ AAEL008940	−2.9433	−3.10494	0.161641	0.996905
AAEL009614/ AAEL009615	−1.81547	−2.35038	0.534903	0.970015

The likelihoods of test and null models (dependence or independence models, respectively) and the *p*-values of log likelihood ratio tests are shown.

By comparisons with predicted orthologous genes among sequenced arthropod genomes (OrthoDB5, http://cegg.unige.ch/orthodb5), we identified a total of 196 overlapping gene pairs of *A*. *aegypti* where at least one gene of each pair was also present among the other seven insect species. But only 19 of these gene pairs in *A*. *aegypti* had both the genes present as orthologs in all the other seven species (Additional file 4). To further confirm that overlapping or non-overlapping localization of genes has no correspondence with presence or absence of orthologs across genomes, we performed hierarchal cluster analysis among the above 19 orthologous gene pairs across the eight species (Additional file 5). The potential for correlation between gene orthology and gene positional overlapping was assessed for statistical significance by Mantel test (see Methods). The correlation was evaluated between binary data of orthologous genes in matrix forms (presence or absence of overlap) with the presence or absence of orthology of the gene pairs. The results showed non-significant correlation between the two (*p* > 0.8) suggesting that gene orthology has no relationship with overlapping localization of genes across species.

### Role of selection on rearrangement of overlapping genes

Another hypothesis about overlapping genes is that mutations occurring within the shared region of overlapping gene pairs would mostly be negatively selected because such mutations may affect adaptation [37] and function of both genes [38,39]. If this is true, we expect to see a higher frequency of synonymous (dS) changes than non-synonymous (dN) changes between orthologous genes when they are present in overlapping manner. To test this expectation, the numbers of per site synonymous and non-synonymous changes in 19 orthologous gene pairs (Additional file 4) were determined among *A*. *aegypti*, *C*. *quinquefasciatus*, *A*. *gambiae*, *D*. *melanogaster* and *P*. *humanus*. The dS and dN values are the rate of synonymous and non-synonymous changes, respectively, between *A*. *aegypti* gene and the corresponding ortholog of other species mentioned above. As shown in Additional file 4, each of these gene pairs is localized in overlapping manner in *A*. *aegypti*. But, the orthologous genes in the other species are found either in overlapping or non-overlapping manner. A binary logit model was developed to fit the data of dS and dN values with the occurrence or non-occurrence of positional overlapping between genes among the species. The dependent variable assumed a value 1 when the genes were found in overlapping position but 0 when the gene pairs were in non-overlapping position in the genome. We performed generalized linear model fitting of the data that is represented by *y* = *β*_0_ + *Xβ* + *e*, where y = dependent variable (overlapping/ non-overlapping of genes), *X* = dS and dN values of both genes, β = coefficient of independent variable, β_0_ represents the value of y when the predictor is equal to zero, and e (error) is assumed to be independent of *X* and has a standard logistic distribution with mean zero. The dS and dN values of both genes (gene1 and gene2) of *A*. *aegypti* gene pairs, calculated by aligning the codon sequence to the orthologs of other insect species (Additional file 4), were used as the independent variables. The results of the regression analysis are shown in Table 3. It shows estimates of coefficients of each of the four independent (predictor variables) in fitting the model to explain the occurrence of the dependent (predicted) variable (i.e. y = 1 or genes are in overlapping position). The estimated regression coefficient shows variation (%) of the outcome with unit change in the predictor variable [because probability (p) of the outcome in the logit model is estimated as the logarithm of the odds {p/(1 – p)}]. The data in Table 3 shows that the coefficients of regression are positive for synonymous changes but negative for non-synonymous changes indicating differential effects of synonymous and non-synonymous mutations on overlapping localization between genes. However, the effects of dS or dN are statistically non-significant in each case (Table 3) indicating that, in these insect species (Additional file 4), the association of synonymous or non-synonymous mutations with overlapping localization of orthologous genes may be a random event. However, the lack of significance may also be due to differences in the reading frames of orthologous genes. Such differences are known to be associated with bias in codon phases of overlapping prokaryotic genes [40]. However, we have not determined from this study if there is a bias in codon phase distribution of overlapping genes that may influence the rate of synonymous and non-synonymous changes between orthologs.

**Table 3:** **Binary logit regression coefficients of rate of synonymous (dS) and non-synonymous (dN) mutations between *A***. ***aegypti *overlapping genes and their 1-to-1 orthologs in other selected insects** (***A***. ***gambiae***, ***C***. ***quinquefasciatus***, ***D***. ***melanogaster *and *P***. ***humanus***)

	**Coefficient**	**Std**. **error**	***p***-**value**
Gene1_dS	0.002	0.000975	0.84
Gene1_dN	−0.838	0.065533	0.61
Gene2_dS	0.009	0.000099	0.39
Gene2_dN	−4.533	0.346861	0.05

The regression was performed in relation to presence or absence of positional overlapping of the orthologous gene pairs across species (see Additional file 4).

### Association of microsatellites with gene overlapping

**Figure 6:**
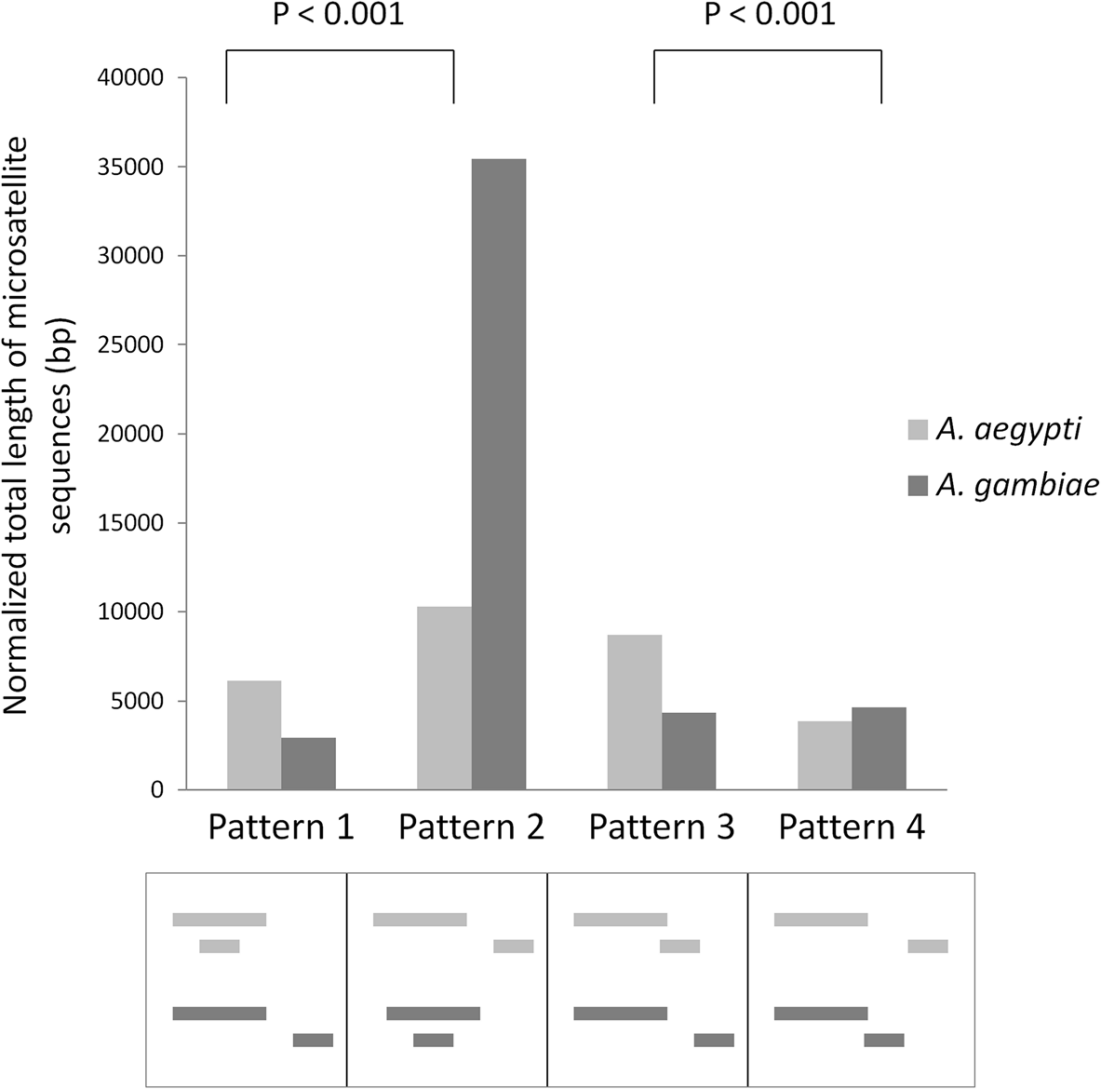
**Association of microsatellites with retention or rearrangement of gene pairs between *A. aegypti *and *A. gambiae.*** Y-axis shows the normalized amount of microsatellite sequences in the gene pairs and X-axis shows patterns (pattern 1 through 4) of overlapping or non-overlapping between the two species. Statistical significance of association of microsatellites between patterns 1 and 2 and between patterns 3 and 4 is shown.

It is well recognized that transposition events contribute to positional rearrangement of genes in eukaryotes [5-7]. And as transposons are known to be intimately associated with simple sequence repeat elements (also known as microsatellites) [41-45], we hypothesized that microsatellites may have a role in positional overlapping of genes. Thus, one of our aims was to determine if there was a significant association between microsatellite contents with rearrangement of overlapping gene pairs between *A*. *aegypti* and *A*. *gambiae*. The amounts (total base pairs) of microsatellite sequences were normalized based on length of shared sequences between E/H gene pairs and their rearranged orthologous pairs in *A*. *aegypti* and *A*. *gambiae*. The results of the 2x2 contingency tests of these data show that positional rearrangement of E/H gene pairs is significantly associated with the amount of microsatellite sequences within the orthologous genes in the two mosquitoes (Figure 6). One scenario is that the repeat sequences, represented as common motifs between the two genes (Additional file 6), are involved in gene rearrangements possibly by facilitating cross over events associated with exchange of the flanking regions between microsatellites [46,47] that lead to positional rearrangements of genes.

### Role of introns in positional overlapping of genes

In *A*. *aegypti* and *A*. *gambiae*, most of the embedded genes (~ 87%) are localized within introns of host genes. Thus, intron loss/gain could contribute to gene relocalization. In that case, we expect that intron loss/gain between one-to-one orthologous genes should be significantly associated with rearrangement of overlapping gene pairs between the two mosquito species. To determine if introns have an association with retention/rearrangement of overlapping genes between the two species, the number of introns among orthologous gene pairs listed in Table 1 were quantified. Based on count statistics of introns between one-to-one orthologous gene pairs between *A*. *aegypti* and *A*. *gambiae*, we found that that loss/gain of introns is significantly (*p* < 0.0001) associated with retention or rearrangement of overlapping gene pairs between the two mosquitoes (Table 4). The rearranged genes show significant loss of introns compared to the orthologous gene pairs located in overlapping positions and *vice versa* suggesting that introns may have a role in gene overlapping and rearrangement. Although intron-mediated gene recombination [48] and references therein or other mechanisms such as intron-transposition [49-51] may be likely mechanisms for these processes, further investigations are needed to determine the exact role of introns in positional overlapping of genes.

**Table 4:** **Significant association of introns with rearrangement of overlapping gene pairs between *A***. ***aegypti*** (**Aaeg) and *A***. ***gambiae*** (**Agam**)

**Gene pair structure**	**Aaeg Gene1** + **Gene2**	**Agam Gene1** + **Gene2**	**Yates Chi square**	**Two tailed *p***-**value**
Non-overlapping in Aaeg but E/H in Agam	694	1056	31.29	p < 0.0001
E/H in Aaeg but non-overlapping in Agam	366	336		
P/O in Aaeg but non-overlapping in Agam	1023	784	16.51	p < 0.0001
Non-overlapping in Aaeg but P/O in Agam	440	471		

### Expression of overlapping genes

Overlapping expression of more than one gene is well known in eukaryotes [33], [52]. We analyzed the expressed sequence tags (ESTs) datasets of *A*. *aegypti* and *A*. *gambiae* to determine if overlapping gene pairs may have overlapping transcripts. Using reciprocal BLASTN searches, we identified several ESTs of *A*. *aegypti* that represented the likely transcription product of overlapping gene pairs (Additional file 7). Although many of these gene pairs are oriented in opposite direction to each other, ESTs were also observed for gene pairs oriented in same direction. Whether these gene pairs are co-transcribed or co-regulated by common upstream/downstream sequences [52] are not known from this study. However, identification of ESTs of overlapping gene sequences clearly shows that these sequences are expressed. Moreover, we show that the annotation of overlapping genes is unaffected whether good evidence of expression (such as EST evidence) is available or not. The dataset of overlapping genes was also analyzed based on availability or non-availability of EST evidence. We found no significant difference in the number of genes that localized in positionally overlapping manner between the two groups (Additional file 8). In *A*. *gambiae*, the EST dataset didn’t reveal such transcripts except for a single gene pair. Although transcripts of overlapping genes were not available in the EST collections of *A*. *gambiae*, we found evidence of expression of these genes (Additional file 9) from published microarray data [24].

Generally, overlapping transcripts are processed by post-transcriptional events to produce individual transcripts of the genes [52]. To assess the expression level of individual gene transcripts of overlapping gene pairs, we examined the microarray expression data of *A*. *aegypti*[23]. Because overlapping genes are predominantly localized in opposite orientation to each other in the genome (Figure 1), we compared expression level of gene pairs (E/H genes) which are either oppositely oriented or oriented in same direction to each other. It was found that the expression levels of overlapping genes in opposite orientation lack significant correlation, whereas the overlapping genes which are oriented in the same direction to each other show statistically significant correlation (*p* < 0.01) (Additional file 10). Most of these genes code for known proteins and have been annotated with start and stop codons suggesting that these genes are not annotation artifacts, although a few genes were annotated as hypothetical proteins. Nevertheless, these results suggested that when the two genes are localized in overlapping manner and also oriented in the same direction, their expression may be co-regulated leading to similar transcription levels. On the other hand, when the two genes are localized in overlapping manner, but oriented in the opposite direction, their transcripts may have differential regulation. In fact, it is well documented that overlapping genes when transcribed in the opposite directions, give rise to sense-antisense transcript pairs which are differentially regulated to play a role in a variety of processes, including mRNA splicing and stability, RNA editing, genomic imprinting and control of translation [33 and references therein].

## Conclusions

The results from this study provide insight into the common prevailing theories of origin and evolution of positionally overlapping genes. These are particularly important for better understanding of distribution and structure of overlapping genes in the genomes of *A*. *aegypti* and *A*. *gambiae*. The genome sequences of both *A*. *gambiae* and *A*. *aegypti* contain gaps that could affect our estimates of overlapping genes in the genome assemblies, but we find this unlikely based on our observation that the overlapping genes are distributed throughout the genome in each species without any bias to specific chromosomal region of *A*. *gambiae* or specific supercontigs of *A*. *aegypti* (data not shown). Furthermore, our estimated frequencies of overlapping genes in mosquitoes are within the range of overlapping gene frequencies reported in other eukaryotes [32,33]. Thus, it is unlikely that there may be large numbers of genes missing because of gaps in sequencing that are positionally overlapping. Nevertheless, the dynamic patterns of positional rearrangement of overlapping genes suggest that these genes may have important roles in genome evolution of vector mosquitoes. Importantly, the information from this investigation may help us in further studies pertaining to evolution and functional characterization of antisense transcripts among overlapping genes in mosquitoes.

## Availability of supporting data

The manuscript is accompanied with the following listed Additional files in the form of supporting data for this study.

## Competing interests

The authors declare that they have no competing interests.

## Authors' contributions

Conceived and designed the experiments: SKB. Analyzed the data: SKB. Contributed reagents/materials/analysis tools: SKB, DWS. Wrote the paper: SKB, DWS. Both the authors read and approved the final manuscript.

## Authors' information

SKB is a Research Assistant Professor in the Department of Biological Sciences and the Eck Institute for Global Health at the University of Notre Dame, Indiana. He has a broad interest in insect genomics and evolution with emphasis on disease transmitting vector species. DWS is a Professor of Biological Sciences and the Director of Eck Institute for Global Health at the University of Notre Dame, Indiana. His work focuses on genetic and genomic analysis of mosquito vector competence to various pathogens as well as on development and application of molecular tools to investigate population biology of mosquitoes.

## Supplementary Material

Additional file 1 **List of E/H and P/O gene pairs of *A*. *aegypti *and *A*. *gambiae.*** Click here for file

Additional file 2 **Overlapping and non-overlapping patterns of orthologous genes between *A. aegypti *and *A. gambiae.*** Click here for file

Additional file 3 **List of gene pairs showing overlapping or non-overlapping localization in *A. aegypti *and *A. gambiae *genomes but have no changes in exon structure.** Click here for file

Additional file 4 **List of one-to-one orthologous genes of *A. aegypti *overlapping genes in other insect species and comparison of overlapping patterns across genomes.** Click here for file

Additional file 5 **Comparison of cluster patterns of retention or rearrangement of gene overlapping (left) with that of presence or absence of orthology of the corresponding gene pairs (right) among different insects.** Red color indicates presence and black color indicates absence of overlapping/ orthology between genes. Click here for file

Additional file 6 List of common microsatellite motifs associated with the overlapping gene pairs that are rearranged between the two mosquitoes. Click here for file

Additional file 7 **List of gene pairs and overlapping ESTs in *A. aegypti.*** Click here for file

Additional file 8 Number of gene pairs identified in positionally overlapping patterns with or without evidence of expressed sequence tags. Click here for file

Additional file 9 **Expression level of gene pairs that are localized in positionally overlapping manner in *A. gambiae* genome.** Click here for file

Additional file 10 **Expression level of *A. aegypti *overlapping genes.** Click here for file
