# Supplementary figures and images for: Overlapping genes of Aedes aegypti: evolutionary implications from comparison with orthologs of Anopheles gambiae and other insects
Source: BMC Evol Biol. 2013 Jun 18;13:124. doi: 10.1186/1471-2148-13-124 (PMC3689595; doi:10.1186/1471-2148-13-124)

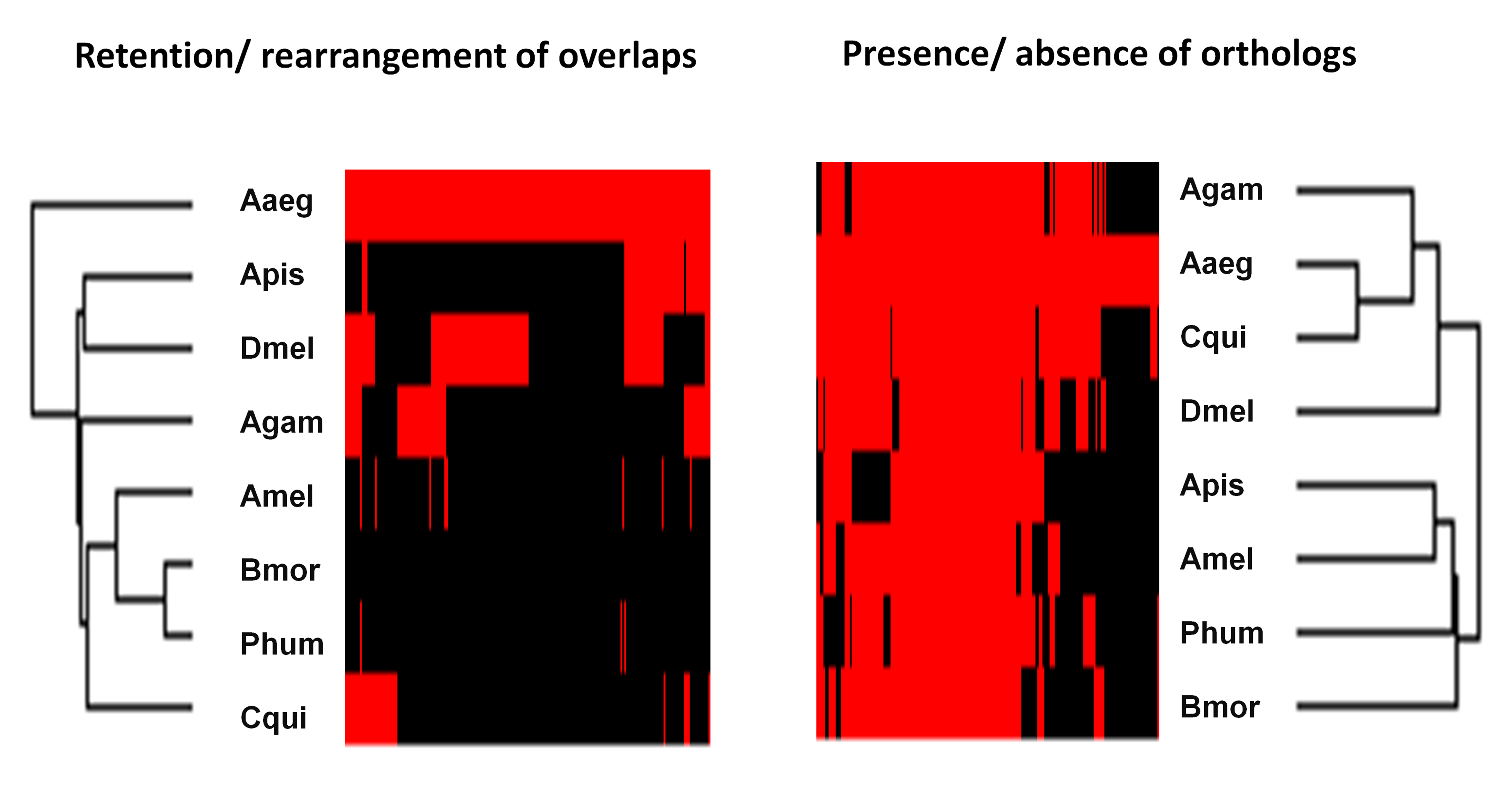

Supplement: Additional file 5 — Comparison of cluster patterns of retention or rearrangement of gene overlapping (left) with that of presence or absence of orthology of the corresponding gene pairs (right) among different insects. Red color indicates presence and black color indicates absence of overlapping/ orthology between genes. [file 1471-2148-13-124-S5.tiff]
