# Supplementary material for: Overlapping genes of Aedes aegypti: evolutionary implications from comparison with orthologs of Anopheles gambiae and other insects
Source: BMC Evol Biol. 2013 Jun 18;13:124. doi: 10.1186/1471-2148-13-124 (PMC3689595; doi:10.1186/1471-2148-13-124)
Supplement: Additional file 7 — List of gene pairs and overlapping ESTs in A. aegypti. [file 1471-2148-13-124-S7.docx]

List of gene pairs and overlapping ESTs in *A. aegypti*. The transcript is identified from available ESTs (expressed sequence tags) by sequence homology searches with the gene pairs.

| Gene pair | Overlapping pattern | Orientation | Overlapping EST | % identity gene pair locus and EST |
| --- | --- | --- | --- | --- |
| AAEL000152 / AAEL000126 | Embedded/Host Gene Pair | Opposite | DV293704 | 99.88 |
| AAEL000167 / AAEL000197 | Embedded/Host Gene Pair | Opposite | DV350936 | 99.88 |
| AAEL000367 / AAEL000346 | Embedded/Host Gene Pair | Opposite | DV296481 | 99.61 |
| AAEL000408 / AAEL000411 | Partially Overlapping Gene Pair | Opposite | DV342704 | 99.56 |
| AAEL000699 / AAEL000720 | Partially Overlapping Gene Pair | Opposite | DV254308 | 99.89 |
| AAEL001775 / AAEL001756 | Partially Overlapping Gene Pair | Opposite | DV356448 | 99.88 |
| AAEL001804 / AAEL001801 | Partially Overlapping Gene Pair | Opposite | DV267181 | 99.87 |
| AAEL003323 / AAEL003304 | Embedded/Host Gene Pair | Opposite | DV368302 | 99.42 |
| AAEL003677 / AAEL003651 | Embedded/Host Gene Pair | Opposite | DV247451 | 99.89 |
| AAEL004192 / AAEL004171 | Embedded/Host Gene Pair | Opposite | DV253765 | 99.88 |
| AAEL004327 / AAEL004329 | Partially Overlapping Gene Pair | Opposite | DV363964 | 99.87 |
| AAEL004498 / AAEL004501 | Embedded/Host Gene Pair | Same | DV434390 | 99.85 |
| AAEL005235 / AAEL005217 | Partially Overlapping Gene Pair | Opposite | DV310172 | 99.58 |
| AAEL005398 / AAEL005388 | Embedded/Host Gene Pair | Opposite | DV339923 | 99.76 |
| AAEL005741 / AAEL005731 | Partially Overlapping Gene Pair | Same | DV312752 | 99.84 |
| AAEL005848 / AAEL005870 | Partially Overlapping Gene Pair | Opposite | DV305801 | 99.87 |
| AAEL006972 / AAEL006963 | Partially Overlapping Gene Pair | Opposite | DV316118 | 99.61 |
| AAEL007157 / AAEL007163 | Partially Overlapping Gene Pair | Opposite | DV329813 | 99.88 |
| AAEL007338 / AAEL007329 | Embedded/Host Gene Pair | Opposite | DV364452 | 99.86 |
| AAEL007567 / AAEL007565 | Embedded/Host Gene Pair | Opposite | DV379958 | 99.89 |
| AAEL007573 / AAEL007581 | Partially Overlapping Gene Pair | Opposite | DV327047 | 99.89 |
| AAEL007867 / AAEL007863 | Embedded/Host Gene Pair | Opposite | DV391068 | 99.73 |
| AAEL008319 / AAEL008330 | Partially Overlapping Gene Pair | Opposite | DV355223 | 99.7 |
| AAEL008341 / AAEL008342 | Partially Overlapping Gene Pair | Opposite | DV299089 | 99.87 |
| AAEL009293 / AAEL009282 | Embedded/Host Gene Pair | Opposite | DV271874 | 99.88 |
| AAEL009984 / AAEL009981 | Embedded/Host Gene Pair | Opposite | DV303243 | 99.59 |
| AAEL010566 / AAEL010559 | Embedded/Host Gene Pair | Opposite | DV237185 | 99.78 |
| AAEL011156 / AAEL011158 | Partially Overlapping Gene Pair | Opposite | DV382369 | 99.88 |
| AAEL012109 / AAEL012104 | Partially Overlapping Gene Pair | Opposite | DV296010 | 99.88 |
| AAEL012244 / AAEL012246 | Embedded/Host Gene Pair | Opposite | DV332186 | 99.88 |
| AAEL013167 / AAEL013169 | Partially Overlapping Gene Pair | Opposite | DV247120 | 99.89 |
| AAEL013232 / AAEL013237 | Partially Overlapping Gene Pair | Opposite | DV297093 | 99.88 |
| AAEL013400 / AAEL013389 | Partially Overlapping Gene Pair | Opposite | DV327623 | 99.89 |
| AAEL013904 / AAEL013902 | Partially Overlapping Gene Pair | Opposite | DW202341 | 99.85 |
