# Supplementary material for: Overlapping genes of Aedes aegypti: evolutionary implications from comparison with orthologs of Anopheles gambiae and other insects
Source: BMC Evol Biol. 2013 Jun 18;13:124. doi: 10.1186/1471-2148-13-124 (PMC3689595; doi:10.1186/1471-2148-13-124)
Supplement: Additional file 8 — Number of gene pairs identified in positionally overlapping patterns with or without evidence of expressed sequence tags. [file 1471-2148-13-124-S8.docx]

Number of gene pairs identified in positionally overlapping patterns with or without evidences of expressed sequence tags.

| Pattern | EST evidence | No EST evidence |
| --- | --- | --- |
| Embedded_opposite orientation | 14 | 314 |
| Embedded_same orientation | 1 | 38 |
| Partial overlapping_opposite orientation | 18 | 334 |
| Partial overlapping_same orientation | 1 | 41 |
